# Supplementary material for: Are carbon emissions trading and green financial instruments synergistic? -Comprehensive quantitative research based on content analysis
Source: PLoS One. 2024 Mar 7;19(3):e0298601. doi: 10.1371/journal.pone.0298601 (PMC10919601; doi:10.1371/journal.pone.0298601)
Supplement: S1 Data — (ZIP) [file pone.0298601.s001.zip › Data/地级市Z_F工作报告.zip!/╕ú╜¿/┴·╤╥╩╨/└┤╫╘╣1⁄2╓┌║┼í╛┬φ┐╦╩2╛▌═°í┐_2006.docx]

——2006年2月7日在龙岩市第二届人民代表大会第七次会议上

各位代表：

　　现在，我代表龙岩市人民政府向大会作政府工作报告，请各位代表连同《龙岩市国民经济和社会发展第十一个五年规划纲要（草案）》一并审议，并请市政协委员和各位列席人员提出意见。

　　2005年和“十五”发展回顾

　　2005年是龙岩发展历史上不平凡的一年。在省委、省政府和市委的正确领导下，我市各级政府坚持以邓小平理论和“三个代表”重要思想为指导，树立和落实科学发展观，认真执行中央、省委和市委的各项决策部署，围绕建设闽粤赣边联结沿海、拓展腹地的生态型经济枢纽，成为海峡西岸经济区重要增长极、集聚极、辐射极的发展定位，以开展保持共产党员先进性教育活动为动力，紧紧依靠全市人民，攻坚克难，乘势而上，全市经济社会保持了持续、协调、健康发展的良好势头，主要指标是近年来完成最好的一年，各项工作取得了新的成绩。

　　初步统计，2005年全市生产总值385.2亿元，增长11.3%，其中第一产业81亿元、增长5%，第二产业174.6亿元、增长16%，第三产业129.6亿元、增长10.1%；农林牧渔业总产值133.5亿元，增长5.5%；全社会固定资产投资123.7亿元，增长34.7%；财政总收入61.1亿元、增长26.7%，其中地方级财政收入24亿元、增长32.9%；实际利用外资1.56亿美元，增长71%；社会消费品零售总额128.7亿元，增长13.4%；城镇居民人均可支配收入10596元、增长9%，农民人均纯收入4125元、增长9.4%。一年来的主要工作和成效是：

　　1、“三农”工作不断加强。认真落实对“三农”的扶持政策，积极促进农业增效、农民增收、农村稳定。全市免征农业税及其附加和除烟叶外的农业特产税及其附加5500万元；发放粮食生产直接补贴625万元，粮食总产113.9万吨；基本完成集体林权制度改革，造林更新面积增长1.1倍；选聘村级农民技术员5475名；组织开展农村劳动力技能培训2.2万人，转移就业7.6万人。全市收购烤烟59.5万担，上等烟比例达43.9%；出栏生猪509.7万头，增长8.9%。全市新增3个农产品获绿色食品标志使用权、12个农产品获无公害食品认证；发展农业专业合作组织和农产品行业协会83个；新增年产值1000万元以上的农业产业化龙头企业6家；全市实现农产品加工产业产值44.1亿元，增长11.7%。完成乡镇通村道路路面硬化1200公里；整理土地1.8万亩，新增耕地1.1万亩；农村“六千”水利工程超额完成省下达计划任务。

　　2、工业和服务业较快发展。实现全部工业总产值386.6亿元、增长17.4%，工业增加值占GDP的比重达到38.6%、提高2.1个百分点；净增规模以上工业企业164家、累计645家，新增产值亿元以上工业企业12家、累计43家，实现规模以上工业总产值293.9亿元、增长22.4%；新增中国驰名商标3件、省著名商标3件。全市获批工业用地1.4万亩，新建标准厂房57.8万平方米；8个工业园区新开工企业112家，增长53.4%。全市规模以上工业经济效益综合指数222.2，居全省首位；地方级财政收入中企业所得税完成1.8亿元，增长54.2%。全市旅游接待389万人次、总收入35亿元，分别增长40%和32.7%；完成房地产投资19.1亿元，增长33.5%；商贸、电信、交通运输、金融、保险等行业持续发展。

　　3、项目带动战略成效显著。“三个一百”项目中，168个投资项目完成投资97亿元，增长49.8%；143个新增长点项目新增产值56.6亿元，增长46.9%；51个在建、新开工重点项目完成投资63.4亿元，增长74.7%，重点项目投资占固定资产投资的比重提高11.8个百分点，工业项目投资占重点项目投资的比重提高2.9个百分点。赣龙铁路建成运营，龙长高速公路基本实现路基全线单幅贯通，龙岩至北京“海西号”快速旅客列车顺利开行，坑口火电厂一期第一二号机组、紫金山万吨级铜矿开发一期、龙马环卫专用车生产线、闽兴专用汽车挂车等一批项目建成投产，龙工装载机技改扩建、马坑铁矿二期技改、卓龙炼钢技改配套、紫金高精度铜及铜板带材、永定国产实业和漳平红狮年产300万吨新型干法水泥生产线、长汀纺织基地、连城罗坊银矿开发、武平高科镁业镁合金生产线、新罗森华冷却肉加工等一批生产性项目加快建设，龙厦铁路通过国家立项，永武高速公路、漳平至永定支线高速公路、漳平火电厂技改扩建等项目前期工作顺利推进。全市新开工投资1亿元以上项目57个，其中工业项目33个；新投产投资1000万元以上的生产性项目105个，同比增加近1倍。全市合同利用市外资金113.7亿元、实到市外资金34亿元，分别增长165.9%和46.7%；累计登记私营企业户数和注册资本分别增长11.8%和39.4%。

　　4、中心城市建设步伐加快。城市总体规划修编纲要基本完成，控制性详细规划覆盖面达90%；实施了34个城建重点项目建设，完成投资2.7亿元，其中龙腾南路、登高西路、北二环路一期、双洋路中段、九一路和凤凰路路面重铺、铁山垃圾中转站等项目建成投入使用，龙腾北路等项目即将建成；龙岩大道二期和登高西路两侧征地拆迁取得突破；更新了50部公交车辆。我市获省级园林城市称号。

　　5、社会事业协调发展。实施了36项国家、省科技计划项目，市科技创业园前期工作加快推进。对2.2万名农村贫困学生实行了义务教育“两免一补”政策，完成了96所农村中小学危房改造；新罗、永定“双高普九”通过省级验收；全市高考录取率81%，上本科线人数首次超过万人；龙岩学院综合办学实力进一步增强。扶持15个乡镇卫生院增加医疗设备，培训乡镇医护骨干201名；对2160名乡村医生实行了政府津贴，对3983名“革命五老”人员实行了定额医疗补助；新罗区新型农村合作医疗试点和农村困难家庭医疗救助试点稳步推进。成功举办了第十二届市运会和第七届市老运会。新增205个50户以上自然村通广播电视。全面推行了农村部分计生家庭奖励扶助制度，全市出生人口政策符合率92%，人口自然增长率5.3‰。国土资源规划、保护与开发水平进一步提高。九龙江、汀江和闽江流域水环境污染综合整治扎实推进，完成高速公路沿线和旅游景区周边27家水泥企业粉尘治理。全市下岗失业人员再就业率60%，城镇登记失业率5%；清理建设领域拖欠工程款完成年度计划。提高了企业退休人员养老金标准，养老、失业、医疗、工伤、生育保险覆盖面扩大；全市5.3万户、13万多人享受城乡低保，基本实现应保尽保。启动了2005—2007年扶贫开发整村推进计划，完成“造福工程”搬迁3294人，1729户受灾无房户春节前全部搬入新居。19项为民办实事项目得到较好落实。基本完成第二次全国经济普查。外事侨务、民族宗教、老区民政、档案、地方志、老年人、妇女儿童、残疾人等工作进一步加强。

　　6、民主法制与精神文明建设扎实推进。认真执行人大及其常委会的决议决定，自觉接受人大及其常委会的法律监督和工作监督，支持政协履行政治协商、民主监督和参政议政职能，加强与各民主党派、工商联、无党派人士和人民团体的联系，虚心听取社会各界人士的建议意见。162件市人大代表建议和246件市政协提案已全部办结。认真贯彻国务院《全面推进依法行政实施纲要》，大力推行行政执法责任制，加强政府法制监督，依法办理行政复议，“四五”普法顺利通过省级验收。廉政建设、效能建设、政务公开、审计监督等工作进一步加强。积极开展创建“平安龙岩”活动，被中央综治委授予“2001—2004年度全国社会治安综合治理优秀地市”称号。“安全生产基层基础建设年”活动扎实推进，安全生产事故起数、死亡人数、受伤人数、直接经济损失四项指标全面下降。积极推进富有闽西特色的精神文明建设，群众性精神文明创建活动继续深化。“双拥”和军警民共建活动深入开展，获全国“双拥”模范城称号，国防动员、民兵预备役、人防等工作取得新进展。

　　各位代表，2005年的努力促进了“十五”计划预期目标的实现，也为“十一五”新一轮大发展奠定了坚实基础。初步统计，与2000年相比，全市生产总值由208.7亿元增加到385.2亿元，年均增长9.8%；三次产业比重由26.7:37.5:35.8调整为21:45.3:33.7；全社会固定资产投资由67.7亿元增加到123.7亿元，五年累计404.3亿元，超过1978年到2000年的投资总和；财政总收入由29.3亿元增加到61.1亿元，地方级财政收入由11.6亿元增加到24亿元，均增长1.1倍;社会消费品零售总额由53.2亿元增加到128.7亿元，增长1.4倍。经过“十五”的发展，全市综合实力迈上了一个新台阶，呈现出良好的发展优势、气势、态势和趋势。“十五”发展的成就突出表现在：

　　产业实力明显增强。工业化进程加快，工业总产值由215.7亿元增加到386.6亿元，年均增长14.2%，工业对经济增长的贡献率达35.8%；烟草、机械、钢铁、建材、纺织、电力等主导产业不断壮大，铜产业从无到有、快速增长；规模以上工业企业和产值亿元以上工业企业个数分别增长83%和169%，龙烟、龙净、龙工、紫矿、卓龙、马坑矿业等骨干企业加速成长，新增上市公司3家。农业五大主导产业与五大特色产品更具竞争力，农产品加工率达40%。旅游、商贸等服务业快速发展，成为全省新兴旅游大市和闽粤赣边重要物资集散地。

　　基础设施明显改善。冠豸山机场建成通航，与长三角、珠三角和闽南金三角实现空中相连。龙梅铁路、赣龙铁路的建成打通了我省第三、第四条铁路进出省通道，全市新增铁路206公里、累计390.9公里。高速公路通车里程达到38.1公里。国省道“改善工程”完成路面重铺332公里，县通乡镇道路路面全部实现硬化，乡镇通村道路路面硬化新增3000公里。新增发电能力149万千瓦，电力装机容量达236.6万千瓦。电话交换机容量比“九五”时期增长1.7倍，移动电话用户、互联网用户分别达到92.4万户和14.6万户。农村“六千”水利工程扎实推进，防灾减灾体系进一步健全。全市基础设施与沿海地区全面接轨，交通枢纽地位日益显现。

　　对外开放明显扩大。五年登记注册外商投资企业191家、注册资本3.7亿美元、外方认缴资金3.2亿美元，分别比“九五”时期增长87.2%、93.3%和122.4%；麦当劳、肯德基、菲莫公司等世界500强企业相继进驻我市或与我市企业合作发展。新增127家企业获外贸进出口经营权，外贸出口总额6.1亿美元、比“九五”时期增长3.1倍。内联协作五年合同利用市外资金232.6亿元，实际到资100亿元。创立了“11&#8226;18”投资龙岩项目洽谈会平台。与港澳台侨交流合作继续加强，五年来岩港澳台同胞和华侨、华人超过5万人次，接受海外捐资兴办公益事业7245万元。与友好城市澳大利亚伍龙岗市的相互往来更加紧密，与法国普松西市、巴西诺昂佩素阿市建立了友好联系。龙岩的知名度逐年提高。

　　城乡面貌明显变化。全市城镇化水平达42%，比2000年提高6个百分点。中心城市五年新建占地50亩以上住宅小区8个、主次干道15条、15层以上中高层建筑15座、市政公用设施22项；新建城市道路71.9公里、排水管道84.6公里、公共绿地77.5公顷、垃圾无害化处理场1座，自来水普及率100%，污水处理率68%，垃圾无害化处理率93.4%，人均公共绿地8.4平方米，万人拥有公交车辆10.2标台；城区面积由2000年的25平方公里扩大到2005年的30平方公里，常住人口从23.7万人增加到28.5万人。各县（市）城区、重点镇和示范新村建设稳步推进，国省道沿线环境卫生整治力度加大。城乡人居环境得到较大改善。

　　体制创新明显推进。市、县、乡党政机构改革顺利完成，事业单位清理整顿与改革稳步推进。下放了169项市级经济社会发展审批权限，县域经济活力增强。五年完成181家国有工业企业改制，置换职工身份4.6万人，累计改制面和职工身份置换面分别达到84.2%和96.5%。农村各项改革不断深化，工作机制不断创新，干部驻村和扶贫开发整村推进取得成效。同时，公共财政体制、社会事业、城市公用事业等改革向纵深推进，非公有制经济较快发展，土地、劳动力、资本等生产要素市场基本形成，社会主义市场经济体制基本建立。

　　发展环境明显优化。出台了《龙岩市促进投资与企业发展的规定》等一系列优惠政策。组建了行政服务中心和项目中心，分五批取消了58.1%的市级行政审批事项，市级审批事项立即办理率76%，市级基建项目审批时限缩短三分之二，推行了告知承诺、一审一核、代办审批、网络审批等服务措施。制定了《关于对损害经济发展软环境行为实行效能告诫的暂行规定》，建立了市直部门发展环境评估评议制度和政府系统绩效评估制度，各级各部门服务发展力度加大，服务质量明显提高。根据去年省外资工作领导小组组织的投资环境监测评价，龙岩在全省九个设区市中仅次于厦门居第2位。

　　社会事业明显进步。五年获省科技进步奖25项，新增省级以上高新技术企业9家，创国家级企业技术中心1家、省级4家。组建了龙岩学院、闽西职业技术学院，扩建中小学350所，改造农村中小学危房32万平方米。组建了市疾病预防控制中心、卫生监督所和传染病院，新建了市第一医院病房大楼、传染病院大楼、疾病预防控制中心实验楼等一批卫生设施，实施了乡镇卫生院“百院建设、千人培训”计划，市、县疾病预防控制体系和医疗救治体系基本建成，夺取了抗御“非典”、高致病性禽流感等重大疫情的胜利。建成或基本建成了闽西文化艺术中心、龙岩图书馆、体育公园、体育大厦等文体设施；顺利完成了古田会议旧址群维修保护一期工程建设；成功申办第13届省运会，我市籍运动员在各级各类比赛中五年共获世界冠军26项、全国冠军86项，6人11次打破8项全国纪录，实现奥运会金牌零的突破；广播电视联网乡镇、村比率分别达99%和69%。连续15年完成省下达人口控制计划。连续6年实现耕地占补平衡，221.1万亩基本农田得到有效保护。环境污染治理和生态保护力度加大，环境质量逐步提高。

　　小康步伐明显加快。与2000年相比，全市城镇居民人均可支配收入由6048元增加到10596元，年均增长11.9%；农民人均纯收入由2959元增加到4125元，年均增长6.9%。城镇居民恩格尔系数从42.4%下降为38.3%，人均住房面积从11.6平方米扩大到32.1平方米。五年新增城镇就业9.2万人；新转移农村劳动力29万人，累计70万人。五年实施为民办实事项目64项，办理省、市人大代表建议871件和省、市政协提案1260件，解决了一批关系人民群众切身利益的实际问题。扶贫开发、社会保险、社会救助、社会福利和慈善事业得到加强，社会治安状况进一步改善。

　　“十五”发展取得的成绩来之不易，这是省委、省政府和市委正确领导，全市上下同心协力、奋力拼搏的结果。在此，我代表市人民政府，向在各个领域和岗位上辛勤工作的全市人民，向给予政府工作大力支持与有效监督的人大代表、政协委员和各民主党派、工商联、无党派人士、人民团体、离退休老同志，向为龙岩改革发展稳定做出积极贡献的中央和省驻岩机构、驻岩部队、武警官兵、公安政法干警，向所有关心支持龙岩发展的海内外、港澳台的朋友们，表示衷心的感谢！

　　“十五”的发展，给我们提供了许多宝贵的启示：一是市委确定的建设生态型经济枢纽，成为海峡西岸经济区重要增长极的目标定位，符合中央、省委的要求和我市实际，在全市上下已经形成了广泛共识，在实践中已经取得了明显成效，必须持之以恒深入落实。二是必须把实施项目带动战略贯穿发展始终，不断深化“两个并举、两个大力，三条原则，四条渠道，五项措施”的项目工作思路和机制，以项目集聚要素、扩大投资、改进服务、推动落实。三是必须强化产业支撑，在大力发展“10+3”产业的同时，加快产业结构优化升级，扎实提高经济增长的质量和效益，努力实现更快更好发展。四是必须深化改革，扩大开放，不断增强沿海意识，用发展的办法解决前进中遇到的各种矛盾和问题，增强发展的动力和活力。五是必须以人为本，统筹协调，推进和谐社会建设，在发展中实现好、维护好、发展好人民群众的根本利益。

　　在肯定成绩的同时，我们也清醒地看到，当前我市经济社会发展仍面临不少困难和问题，主要是：经济总量偏小，能带动、支撑全市经济社会持续发展的大项目不多；产业结构不够合理，非资源型项目、龙头企业、终端产品、名牌产品偏少，产业集聚度不高，经济增长方式比较粗放；外向型经济、民营经济总量较小，活力有待增强；城乡区域发展不平衡，农村社会事业发展相对滞后，农民持续增收难度较大，中心城市辐射带动能力较弱；社会就业与保障压力大，部分群众生活还比较困难，安全生产基础仍然比较薄弱，环境污染治理与生态保护需要加大力度；政府部门一些工作人员缺乏责任心、办事效率低、工作落实不到位，机关作风与效能建设有待加强。对上述问题，我们将认真对待，采取强有力的措施，扎扎实实地加以解决。

　　“十一五”时期发展目标与主要任务

　　各位代表，“十一五”时期是我市经济社会发展的重要战略机遇期，也是建设生态型经济枢纽，成为海峡西岸经济区重要增长极的关键时期。党中央、国务院和省委、省政府对闽西老区的发展十分重视和关心。就在“十一五”开局之初，胡锦涛总书记亲临福建考察，第一站就到了龙岩，他对我市经济社会发展取得的成绩给予了充分肯定，并语重心长地说：“老区人民为中国革命的胜利作出了重大贡献，中央将会继续支持老区的建设与发展。你们现在有机场，高速公路通了，铁路也修起来了，加上建设海峡西岸经济区的机遇，为龙岩的发展创造了更好的契机，希望继续抓住机遇，同心协力，艰苦奋斗，把老区发展得更好”。省委卢展工书记要求“龙岩要很好地学习胡锦涛总书记的重要讲话，把老区建设、海西建设结合在一起，让老区在海西建设中发挥重要作用”。胡锦涛总书记的重要讲话和省委卢书记的重要指示，充分体现了党中央、省委对龙岩工作的充分肯定，对闽西老区的极大关心和殷切期望，给予我们巨大的鼓舞和鞭策。站在新的历史起点上展望未来，闽西老区的发展步伐一定会进一步加快，闽西老区的明天一定会更加美好！

　　这次提请大会审议的《龙岩市国民经济和社会发展第十一个五年规划纲要（草案）》（以下简称《纲要》），是根据胡锦涛总书记在福建和我市考察时的重要讲话精神，以及党的十六届五中全会、省委七届十次全会精神和市委《关于制定龙岩市国民经济和社会发展第十一个五年规划的建议》要求，在广泛征求各方面意见、集中全市智慧的基础上制定的。《纲要》总结了“十五”计划的实施情况，分析了“十一五”发展面临的机遇与挑战，提出了今后五年经济社会发展的指导思想、目标任务、保障措施和重大项目。

　　今后五年发展的指导思想是：以邓小平理论和“三个代表”重要思想为指导，全面树立和落实科学发展观，认真贯彻胡锦涛总书记在福建考察时的重要讲话和把龙岩老区发展得更好的重要指示，按照省委提出的“四个推进”、“四个基本”、“四个关键”的战略部署和我市建设生态型经济枢纽、成为海峡西岸经济区重要增长极的目标定位，坚定不移实施项目带动战略，围绕发展抓产业、围绕产业抓项目，围绕项目抓服务、围绕服务造环境，发挥优势、营造气势、呼应态势、持续趋势，扎实推进新一轮大发展，为构建和谐社会，提前实现全面建设小康社会而努力奋斗。

　　发展目标是：生态型经济枢纽初具雏形，海峡西岸经济区重要增长极基本形成，综合实力和经济竞争力显著增强，构建和谐社会取得明显成效。年均经济增长速度比全省平均水平高1—2个百分点，力争到2010年，地区生产总值和财政总收入比2005年翻一番，工业总产值突破1000亿元，资源利用效率显著提高。社会事业协调发展，公共服务和社会管理体系进一步健全，民主法制建设、精神文明建设、社会治安、安全生产取得新进步。

　　今后五年经济社会发展的主要任务是：

　　一是建设社会主义新农村。按照“生产发展、生活宽裕、乡风文明、村容整洁、管理民主”的要求，因地制宜制定和实施社会主义新农村建设规划，围绕推进社会主义新农村建设，坚持“多予少取放活”，建立以工促农、以城带乡的长效机制，加大力度扶持“三农”，继续深化农村改革，发展现代农业，推进农业产业化，加快发展农村经济，加强农村人力资源开发，争取农民人均纯收入年均增长7%以上，协调推进农村经济、政治、文化和社会建设。

　　二是推进产业结构升级。加强龙头带动、平台建设、科技创新、品牌打造，促进产业集聚，壮大“10+3”产业，力争到2010年烟草、机械、钢铁、铜、建材5个产业年产值均达到100亿元以上，纺织、电力、煤炭、农产品加工和旅游5个产业年产值均达到50亿元以上，电子信息、医药化工和物流3个产业加快发展。加快转变经济增长方式，争取到2010年万元GDP综合能耗比“十五”期末下降20%，形成规模与效益、速度与质量相协调的产业支撑体系。

　　三是努力完善基础设施。按照适度超前、功能配套、高效便捷的要求，加快建设包括一个机场、五条高速公路、六条铁路、八条国省道在内的现代综合运输体系，以及能源保障体系、信息基础设施体系和防灾减灾体系。

　　四是促进区域协调发展。突出中心城市龙头带动作用，统筹城镇发展规模、经济布局和基础设施建设，促进人口和生产要素向城镇集聚，推动中心城市、县城和小城镇协调发展，争取到2010年全市城镇化水平达到48%。

　　五是全面推进对外开放。充分发挥区位、资源、产业、环境、文化等发展优势，大力发展外向型经济，推进区域协作，构建以开放促合作、以合作促发展的大开放格局，争取实际利用外资、外贸出口分别年均增长10%和15%，到2010年累计实际利用外资超过10.5亿美元。

　　六是推进社会事业发展。加快发展科教文卫体广电等社会各项事业，争取到2010年全市高新技术企业达25家以上，农村劳动力人均受教育年限达9年以上；群众性文化设施达到国家标准；公共卫生体系建设全面加强，主要健康指标接近世界中等发达国家水平；人均体育场地面积、市民体质合格率达到全省先进水平；广播电视综合覆盖率达99%；人口年均自然增长率控制在9.2‰以内。

　　七是强化生态环境保护。继续实施可持续发展战略，加强生态保护与环境污染治理，争取到2010年全市森林覆盖率稳定在78%以上，水环境功能区达标率达90%，矿产资源综合利用率达30%以上，95%的重点水土流失区得到治理，城镇生活垃圾无害化处理率达70%、生活污水集中处理率达60%，村镇饮用水卫生合格率达90%，二级环境空气达标率达95%。

　　八是深化体制机制创新。坚持社会主义市场经济的改革方向，积极推进各项改革与制度创新，更大程度地发挥市场在资源配置中的基础性作用，加快形成有利于转变经济增长方式、促进全面协调发展的体制机制。

　　九是全面构建和谐社会。坚持以人为本，综合运用法律、行政、经济、政策、道德等手段，有效整合各种社会资源，加快构建民主法治、公平正义、诚信友爱、充满活力、安定有序、人与自然和谐相处的社会。

　　为了促进上述任务的落实，《纲要》提出要深入实施项目带动战略，强化产业政策引导，创新项目工作机制，优化经济发展环境；加快转变政府职能，改进行政管理方式，加强社会管理与公共服务，提高依法行政能力；扎实推进规划实施，加强监督检查，促进各项目标的全面实现。展望“十一五”，我们充满新一轮大发展的信心与决心。

　　2006年主要预期目标和重点工作安排

　　各位代表，2006年是“十一五”的开局之年和推进我市新一轮大发展的关键之年，各项预期目标的安排必须积极稳妥。全市经济社会发展的主要预期目标是：生产总值增长11%，在提高经济增长质量和效益的基础上，力争发展得更快更好些；全社会固定资产投资增长15%；财政总收入、地方级财政收入分别增长13%；实际利用外资增长33%；外贸出口增长30%；社会消费品零售总额增长13%；城镇居民人均可支配收入增长7%，农民人均纯收入增长5%；城镇登记失业率控制在5%以内；人口自然增长率控制在5.5‰以内。为实现上述目标，将重点做好以下六个方面工作：

　　1、坚持以“三农”工作为重中之重，加快建设社会主义新农村。

　　千方百计促进农民增收。认真制定社会主义新农村建设规划，抓好10个乡镇、100个村的试点工作。稳定、完善和强化各项支农政策，加大对“三农”的投入与扶持。围绕提高农业综合生产能力，稳步发展粮食生产，加快做大烤烟、优质茶、早熟水果、食用菌、反季节蔬菜，做强瘦肉型猪和地方优质畜禽等农业优势产业，发展现代林业。加强农业科技服务，推广农业标准化生产，健全农业信息体系、农产品质量安全体系和动植物疫病防控体系，力争新增3个省级以上农业标准示范区、10个“绿标”产品、5个无公害认证产品。完善农产品市场体系，促进农产品销售。推进农村人力资源开发，扶持8万名农村劳动力参加职业技能培训，转移就业6.3万人。

　　大力推进农业产业化。加快新罗森华冷却肉加工、长汀盼盼休闲食品加工生产线、连城地瓜干加工园区二期、漳平龙泰安农产品加工等项目实施进度，加强特色优势农产品加工项目的推介招商，推进闽台农业合作，争取新增市级以上农业产业化龙头企业13家。加快推广“公司+协会+农户”的农业产业化经营模式，积极发展农业专业合作组织和农产品行业协会，支持龙头企业就地建立原料基地、招收本地农民进厂务工，带动农民增收。

　　加强农村基础设施建设。新增乡镇通村道路路面硬化700公里。继续抓好农村“六千”水利工程建设，完成水库除险加固19座、村级供水235处，开工建设小型水库蓄水工程4座、山地水利水池500个、标准化渠道200公里，新增节水灌溉5万亩，治理水土流失18万亩，整治河道13公里；建成九龙江防洪堤6公里，开展水毁水利工程集中整治。改善10万亩烟田基础设施。开展农村“一池三改”户用沼气工程建设。加强以防汛抗旱和森林防火为重点的防灾减灾设施建设，健全农村重大自然灾害预警体系。推进以养殖业污染治理、垃圾无害化处理和旧宅基地整理为重点的农村环境卫生综合整治。

　　加快发展农村公共服务事业。在落实对农村贫困家庭子女义务教育“两免一补”政策的同时，对农村义务教育阶段学生全部免收杂费，完善高校和中等职业学校家庭困难学生助学体系，完成第二期农村中小学危房改造工程。启动县级以上医院挂钩帮扶乡镇卫生院工作，推进实施“百院建设、千人培训”计划，对重点优抚对象和“革命五老”人员实施医疗救助，推进新罗区新型农村合作医疗试点。启动实施“百镇千村”农村文化建设工程，抓好首批20个乡镇、100个村的文化设施建设。实现80%的行政村完整覆盖市、县两级广播电视节目，完成610个20户以上自然村通广播电视。充分发挥农技员、国土资源和规划建设协管员、计划生育管理员、乡村医生、治安调解员等农村“五大员”的作用，全面加强农村公共服务与管理。

　　2、坚持以做大做强“10+3”产业为主导，加快提高经济增长质量和效益。

　　大力发展“10+3”产业。落实“六个一”工作机制，完善产业发展规划，科学制订年度目标及扶持政策，加大协调服务力度，加快推进重大项目，促进产业加快发展。集中各种资源向“10+3”产业中的龙头企业、优势企业倾斜，加强银企合作，做好煤电油运、环境容量、用地等综合平衡，支持龙头企业开展股份制改革和争取上市，促进产业链延伸与产业协作配套。加快工业园区和工业集中区建设，确保“11&#8226;18”前建成200万平方米标准厂房，健全园区配套设施和社会化服务体系，培育产业集群。

　　加快推进新型工业化。重点抓好173项新增产值1000万元以上的工业新增长点项目、70项投资3000万元以上的工业投资项目和100项工业技术创新、新产品开发、电子信息应用项目，继续实行领导挂钩责任制，着力解决项目实施中存在的困难问题，确保工业较快增长。引导企业推进自主创新，帮助有条件的企业建立博士后科研工作站，争取新增国家级企业技术中心1家、省级3家、市级20家。积极实施品牌战略，推行质量标准体系，力争新创一批国家驰名商标、省著名商标和国家、省名牌产品。大力发展循环经济，依靠技术创新，抓好钢铁、有色金属、煤炭、电力、化工、建材等重点行业的节能降耗，推进资源综合利用、精深加工和再生资源循环利用，加快建立资源节约利用的长效机制。

　　积极发展服务业。大力发展旅游业，加强以红色旅游、客家文化旅游、生态旅游为重点的旅游项目建设；完善旅游服务配套开发，力争新开工五星级酒店1家、四星级酒店3家；积极推进土楼申报“世遗”，办好第四届旅游节，加强旅游促销和区域协作，不断拓展旅游市场。加快发展现代物流业，抓好闽西交易城二期、闽西粮油饲料城二三期等市场建设，积极引进大型物流企业，继续发展连锁经营和电子商务。完善外贸支持服务体系和预警监测机制，继续实行外贸出口奖励政策，壮大外贸经营主体，扩大外贸出口规模。稳步发展房地产业，加强供地调控，优化供给结构，激活二级市场，规范物业管理。积极发展金融、信息、商务、中介等现代服务业。

　　3、坚持以实施项目带动战略为主线，加快扩大投资规模。

　　突出抓好重点项目建设。初步安排市重点项目70项，年计划投资68.8亿元。其中在建重点项目41项，年计划投资57.3亿元；预备新开工项目19项，年计划投资11.5亿元；前期工作项目10项，总投资178亿元。初步明确列入省重点项目47项，年计划投资54.6亿元。加快龙长高速公路、坑口火电厂一期第三四台机组、龙工装载机系列项目、紫金高精度铜及铜板带材、永定国产实业和漳平红狮年产300万吨新型干法水泥生产线等在建项目实施进度，开工建设龙厦铁路、永武高速公路和长汀凯鲍专用汽车生产线等项目，完成漳平至永定支线高速公路立项，加快推进坑口火电厂二期、漳平火电厂技改扩建、龙马中重型卡车等项目的前期工作。

　　滚动实施“三个一百”项目计划。初步安排新增长点项目182项，年计划新增产值56.5亿元；投资项目239项，年计划投资108.9亿元；储备项目131项，总投资354亿元。继续对“三个一百”项目实行严格的目标管理，强化领导挂钩责任制、分级管理制、业主负责制和督查联动机制，采取有力措施破解项目推进中遇到的瓶颈制约，落实质量、进度、投资、安全、廉政“五同步”，确保项目顺利推进。

　　全力拓展招商引资。实施“深化港澳台，加强东南亚，拓展欧美日”的招商策略，加强政府组团招商、投资与发展顾问招商、挂职干部招商、协会（商会）招商、产业招商和以商招商，精心组织参加“5&#8226;18”、“6&#8226;18”、“9&#8226;8”等招商和项目对接活动，邀请周边地市联办“11&#8226;18”招商活动，提高利用外资的规模和质量。将重大经贸活动签约项目履约情况列入目标管理考核，建立跟踪推进与协调服务机制，提高项目履约率。主动承接沿海发达地区产业转移，加强闽西南5市、闽粤赣13市和闽粤赣边协作区的区域协作，拓展与厦门市的山海协作和对口帮扶，重视引入回乡创业资金，力争新签内联项目310项，实到市外资金38亿元。

　　大力开发和培育项目源。不断拓展项目生成思路，整合项目开发力量，围绕“十一五”规划纲要、六大支撑体系建设和“10+3”产业发展，重点开发和征集一批生产性项目、非资源型项目、产业链延伸项目、高科技项目，完善配套政策，增强项目吸引力。健全项目中心服务机制，加强项目储备库建设，推进市、县项目资源共享，为企业和投资者提供更加有效的项目推介、咨询和跟踪服务。加快推进“十一五”各个专项规划项目的前期工作。

　　4、坚持以中心城市建设为带动，加快推进城镇化进程。

　　加大中心城市规划建设管理力度。基本完成中心城市总规修编，继续抓好2006—2010年中心城市近期建设规划等专项规划编制。加快龙岩经济技术开发区、新罗民营科技园区和雁石等重点工业集中区的发展。稳步推进行政中心、五星级酒店等带动新区建设项目和5个城市组团建设。继续滚动实施2003—2007年100个城建项目，重点抓好38项城市道路、污水管网、市政改造等项目，加快推进第二水源项目建设。加强监管，严肃查处违法占地和违章建设问题。开展中心城市新一轮环境综合整治工作，稳步实施规划区内矿山和水泥等污染企业的关停并转，继续抓好龙津河污染和燃煤炉灶、建筑施工扬尘和机动车尾气等整治。加快发展城市公交事业。深化城建投融资体制改革，促进公用事业市场化、产业化、社会化运营。

　　推动县域经济更快更好发展。落实市领导挂钩县（市、区）和重点产业工作制度，加强对产业发展、项目建设、重大规划编制等的指导与服务。推进简政放权，鼓励建立有利于县域经济发展的体制机制。加强市、县联动发展，开展产业联合招商，推进产业协作配套，探索工业联合集中发展路子，优化区域发展资源配置。完成市通县公路路面重铺70公里。加大力度扶持欠发达地区发展，努力缩小县域发展差距。继续推进一批示范镇、村建设。

　　5、坚持以体制创新为动力，加快深化各项改革。

　　加快发展民营经济。制定提升民营经济发展水平的政策，健全民营企业信用担保、技术创新、人才培训、市场开拓、对外合作等服务体系，重点在基础设施、市政公用事业以及现代服务业领域推出一批符合民营企业投资取向的项目，激活民营投资，提升民营经济竞争力。

　　深化国有资产管理体制和国有企业改革。按照总体规划、分步授权的原则，将市属国有资产纳入市国资委统一监管。理顺国资委与企业主管部门、国投公司、企业之间的关系，完善国有资产监管制度，确保国有资产保值增值。基本完成市属国有工业企业“两个置换”改革，加强已改制企业的规范管理。

　　创新农村工作机制。巩固农村税费改革成果，深化集体林权制度改革和粮食流通体制改革，稳步推进小型水利设施产权制度改革。新建10个乡镇“三农”服务中心，在128个村开展 “农民之家”试点建设。创新金融支农机制，推广小额信贷和农户联保贷款，推进农村信用户、信用村、信用乡镇创建活动。加快化解乡村债务。依法完成村级组织换届选举。

　　加快财税体制改革。深化部门预算改革，开展国库集中支付改革试点，强化财政监督和预算约束，提高财政资金使用效益。坚持依法治税，加强重点税源监控，加大税收管征和退税监管力度，强化政府非税收入管理。调整优化财政支出结构，重点加大对产业发展与项目建设的支持力度，进一步完善扶持发展的财税政策体系。

　　6、坚持以人为本，加快构建和谐社会。

　　加强生态环境保护和建设。基本完成市级土地利用总体规划修编；认真组织实施市“十一五”矿产资源规划、环境保护规划等专项规划，加强矿产、耕地、森林、水等各类资源的科学保护、开发与管理，推进“青山挂白”治理；加大煤炭等重要矿产资源的勘查投入。加强生态建设，完成造林30万亩，抓好生态功能区管护。加大环境质量监控力度，继续开展大气粉尘、污水、垃圾、噪声等综合治理，抓好九龙江、汀江和闽江流域水环境污染综合整治，加强饮用水源保护。

　　加快发展社会事业。落实《龙岩市科学技术奖励办法》，加快科技创业园一期建设，继续申报实施一批国家、省科技计划项目，鼓励企业与高校、科研院所密切产学研合作。加强人才队伍建设，完善人才市场服务体系，健全激励机制，充分发挥人才对发展的支撑作用。围绕教育均衡发展，扎实推进“双高普九”，促进城乡教育协调发展，更加重视高中教育与幼儿教育，大力发展职业教育和民办教育，加快提高龙岩学院、闽西职业技术学院等高校的办学质量，继续做好申办福建畜牧兽医职业学院的前期工作。健全市、县疾病预防控制体系和医疗救治体系，加强市传染病院和县级医院传染科建设，抓好重大传染病和地方病防治；强化社区卫生服务，将10所县城所在地乡镇卫生院转制为社区卫生服务机构；继续抓好“餐桌污染”治理。认真落实“文化强省”战略，进一步打造红色、客家、河洛文化品牌，抓好古田会议旧址群维修保护二期工程建设，加强文化精品创作，推进文化体制创新，加快发展文化产业。完成市广电大楼一期工程，启动有线数字电视项目。做好承办第13届省运会工作，实现“保五争四”目标。巩固计生工作成果，建立健全人口和计生工作新机制，稳定低生育水平，提高人口素质。

　　着力抓好惠及百姓的实事。加快建立城乡一体的就业工作机制，促进就业再就业，力争新增城镇就业2.3万人、下岗职工再就业1.5万人。加强劳动保障监察执法，积极创建和谐劳动关系企业和工业园区，加快建立解决拖欠农民工工资和建设领域拖欠工程款的保障机制。提高非公企业社会保险参保率，完善企业退休人员社会化管理机制，继续做好城乡低保工作，推行农民住房保险，探索建立失地农民保障机制。扩大城乡困难家庭医疗救助试点范围，加快推进解决群众看病难、看病贵的问题。抓好社区组织换届，推进和谐社区建设。加快扶贫开发整村推进，完成“造福工程”搬迁3000人。继续开展“平安龙岩”创建活动，力争漳平、武平、连城、长汀4县（市）通过省级验收，人民群众对社会治安满意和基本满意率达到90%以上。严格落实安全生产责任制，深入开展专项整治，抓好隐患整改，严肃责任追究，提升管理水平。加强公共应急机制建设，提高预防和处置突发事件的能力，维护公共安全和社会安定。

　　加强精神文明建设。深入开展多种形式的精神文明建设创建与竞赛活动，不断创新活动载体与内容，大力提高市民思想道德素质和社会文明程度，争创省级文明城市。积极推进“诚信龙岩”建设。加强“双拥”、国防动员、民兵预备役、武警、消防和人防工作，抓好统计、外事侨务、民族宗教、老区民政、气象、水文、档案、地方志、老年人、妇女儿童、残疾人等各项工作。

　　全面建设对人民负责的政府

　　各位代表，建设生态型经济枢纽，使龙岩成为海峡西岸经济区重要增长极，是全市人民的共同心愿，也是政府义不容辞的责任。面对新机遇、新形势、新要求，我们将本着对全市人民高度负责的精神，认真履行职责，树立推动工作、对人民负责、公正廉洁的形象，切实担负起人民赋予的重任。

　　转变职能，依法行政。我们要主动接受党委的领导，认真执行人大及其常委会的决议决定，自觉接受人大及其常委会的法律监督、工作监督和政协的民主监督，认真办理人大代表建议和政协提案，虚心听取各民主党派、工商联、无党派人士和人民团体的意见，全面履行政府经济调节、市场监管、社会管理和公共服务四大职能。自觉把依法行政作为政府工作的基本准则，不断提高政府工作人员的法治意识和依法行政水平，严格依照法定权限和程序办事，善于运用法律手段处理各种矛盾和问题，加快健全行政执法体制，推进城市管理综合执法，提高行政复议质量，启动“五五”普法，积极推进依法治市。自觉把转变职能作为依法行政的关键环节，进一步深化行政审批制度改革，减少和规范审批事项，创新政府管理方式，提高为市场主体服务的质量，营造良好发展环境。

　　落实责任，务实勤政。时刻铭记加快闽西老区发展的历史责任，把对组织负责与对人民负责统一起来，对当前负责与对长远负责统一起来，健全以项目为核心的目标管理考评机制和行政责任体系，层层落实责任，一级对一级负责。发扬“平静之中的满腔热血，平凡之中的伟大追求，平常之中极强烈的责任感”的“三平精神”，从基层基础抓起，从解决最突出的矛盾问题抓起，从牵一发动全局的关键环节抓起，真抓实干，说了就做、说到做到、说好做好。把老区艰苦奋斗的实干精神和新时期开拓进取的创新精神结合起来，始终保持奋发有为的精神状态，敢闯敢试，敢于负责，扑下身子做工作，沉下心来干事业，勤勤恳恳，甘于奉献，为闽西新一轮大发展尽职尽责，再立新功。

　　提高效率，从严治政。以贯彻实施《公务员法》为契机，加强公务员教育培训，健全公务员任用奖惩机制，不断提高公务员队伍的整体素质，尤其是各级政府及部门领导干部驾驭市场经济的能力、攻坚克难的能力和处理突发公共事件的能力。落实行政机关效能建设“一把手”责任制，抓好政府及其部门绩效评估工作，深化“加快闽西发展效能督查行”活动，加强政府机关面对群众和企业“第一道门槛”的效能建设，把提高效率体现在研究问题、解决问题上，体现在落实好已经出台的政策措施上，体现在抓好项目生成和组织实施上，切实做到公共服务提供到哪里、绩效评估就延伸到哪里，行政权力运行到哪里、效能监督就推进到哪里。

　　牢记宗旨，廉洁从政。牢记“两个务必”，禀承对人民负责、为人民办事、受人民监督、让人民满意的原则，忠实行使人民赋予的权力，用心关注民生、倾听民意、体察民情、疏解民困，多干群众急需的事，多干能使最广大人民群众受益的事，多打基础，多谋远虑，不搞花架子，不耍嘴皮子，把执政为民的要求落实到政府各项工作之中。严格执行廉洁自律各项规定，认真落实党风廉政建设责任制，加快健全教育、制度、监督并重的惩治和预防腐败体系。规范“用权、理财、管人”行为，完善建设工程招投标、经营性土地使用权出让、国有产权交易、政府采购等制度，强化行政监察和审计监督，推行政务公开。切实治理部门和行业不正之风，严厉查处违法违纪案件。

　　各位代表，宏伟的目标正激励着我们奋勇向前。让我们紧密团结在以胡锦涛同志为总书记的党中央周围，高举邓小平理论伟大旗帜，深入贯彻“三个代表”重要思想，全面落实科学发展观，在中共龙岩市委的领导下，团结和依靠全市人民，立足新起点，把握新机遇，迎接新挑战，创造新业绩，共同谱写闽西老区更快更好发展的新篇章！
